# A putative nuclear copper chaperone promotes plant immunity in Arabidopsis

**DOI:** 10.1093/jxb/eraa401

**Published:** 2020-08-31

**Authors:** Long-Xiang Chai, Kai Dong, Song-Yu Liu, Zhen Zhang, Xiao-Peng Zhang, Xin Tong, Fei-Fan Zhu, Jing-Ze Zou, Xian-Bing Wang

**Affiliations:** 1 State Key Laboratory of Agro-Biotechnology, College of Biological Sciences, China Agricultural University, Beijing 100193, China; 2 University of Edinburgh, UK

**Keywords:** Copper chaperone, plant immunity, PR1, *Pseudomonas syringae* pv *tomato* DC3000, salicylic acid, TGA2

## Abstract

Copper is essential for many metabolic processes but must be sequestrated by copper chaperones. It is well known that plant copper chaperones regulate various physiological processes. However, the functions of copper chaperones in the plant nucleus remain largely unknown. Here, we identified a putative copper chaperone induced by pathogens (CCP) in *Arabidopsis thaliana*. CCP harbors a classical MXCXXC copper-binding site (CBS) at its N-terminus and a nuclear localization signal (NLS) at its C-terminus. CCP mainly formed nuclear speckles in the plant nucleus, which requires the NLS and CBS domains. Overexpression of CCP induced PR1 expression and enhanced resistance against *Pseudomonas syringae* pv. *tomato* DC3000 compared with Col-0 plants. Conversely, two CRISPR/Cas9-mediated *ccp* mutants were impaired in plant immunity. Further biochemical analyses revealed that CCP interacted with the transcription factor TGA2 *in vivo* and *in vitro*. Moreover, CCP recruits TGA2 to the *PR1* promoter sequences *in vivo*, which induces defense gene expression and plant immunity. Collectively, our results have identified a putative nuclear copper chaperone required for plant immunity and provided evidence for a potential function of copper in the salicylic pathway.

## Teaser

A new putative copper chaperone localizes in the nucleus and improves plant defense response in *Arabidopsis thaliana*.

## Introduction

Copper ions are cofactors for many enzymes in biological processes including electron transport, hormone perception, and free radical elimination (Pilon *et al.*, 2006; Robinson and Winge, 2010). Copper exists as Cu^2+^ or Cu^1+^, between which redox cycling triggers production of toxic hydroxyl radicals and severe damage to lipids, proteins, DNA, and other biomolecules (Robinson and Winge, 2010). Thus, copper chaperones, a set of soluble copper-binding proteins, sequestrate copper and limit the exposure of free copper to the cytoplasm. Moreover, copper chaperones are responsible for intracellular copper trafficking to their specific target copper proteins or compartments (Harrison *et al.*, 1999). Copper chaperones usually harbor a classical MXCXXC metal-binding motif that is required for their physiological functions (Harrison *et al.*, 1999).

The Arabidopsis genome encodes seven copper chaperones, namely the copper chaperone for superoxide dismutase (CCS), antioxidant protein1 (ATX1), ATX1-like copper chaperone (CCH), cytochrome *c* oxidase 11 (COX11), COX17, and two homologs of the yeast copper chaperone (HCC1 and HCC2) (Himelblau *et al.*, 1998; Chu *et al.*, 2005; Puig *et al.*, 2007; Burkhead *et al.*, 2009; Attallah *et al.*, 2011). In Arabidopsis, CCS delivers copper to Cu/Zn superoxide dismutases (SODs) in the cytoplasm, chloroplast, and peroxisome, respectively (Kliebenstein *et al.*, 1998; Burkhead *et al.*, 2009). ATX1 and CCH share high sequence homology with the yeast ATX1 protein and both can complement the yeast *atx1* mutant, but they have distinct properties and functions in copper homeostasis (Puig *et al.*, 2007; Shin *et al.*, 2012). ATX1 contributes to tolerance of both excess and deficient copper through its copper-binding MXCXXC motif (Shin and Yeh, 2012; Shin *et al.*, 2012). In contrast, the biological role of CCH in the copper homeostasis process has not been well defined (Shin and Yeh, 2012; Shin *et al.*, 2012). In addition, copper chaperones COX11, COX17, HCC1, and HCC2 play important roles in mitochondrial respiration (Balandin and Castresana, 2002; Burkhead *et al.*, 2009; Attallah *et al.*, 2011). Although copper chaperones have been extensively studied, much information is still lacking, particularly concerning whether copper chaperones and copper are imported into the nucleus and mediate plant defense responses.

Salicylic acid (SA), one of the major plant hormones, plays a central role in various plant immune response to pathogens, including pathogen-associated molecular pattern (PAMP)-triggered immunity (PTI), effector-triggered immunity (ETI), and systemic acquired resistance (SAR) (Seyfferth and Tsuda, 2014; Yan and Dong, 2014). SAR is an SA-induced broad-spectrum defense mechanism, in which the non-expressor of pathogenesis-related gene 1 (NPR1) is a key regulator (Cao *et al.*, 1994; Fu and Dong, 2013). As the SA concentration increases upon pathogen infection or exogenous application of SA, oligomerized NPR1 is reduced to monomers in the cytosol and translocated into the nucleus. and mediates transcriptomic changes (Kinkema *et al.*, 2000; Mou *et al.*, 2003; Wang *et al.*, 2006). Since it lacks a known DNA-binding domain, NPR1 regulates *pathogenesis-related* (*PR*) gene expression through interaction with nuclear transcription factors, such as TGAs and NIMINs (Zhang, 1999; Després *et al.*, 2000; Weigel *et al.*, 2001). The expression of *PR* genes is induced by SA, which is used as the best understood readout for SAR (Uknes *et al.*, 1992). The induction of the *PR1* gene by SA is dependent on the presence of the activation sequence-1 (as-1) element in the promoter regions of the *PR1* gene (Qin *et al.*, 1994). In healthy cells, the as-1 element specifically binds to the TGA transcription factors, leading to repressed transcription of *PR1* under a normal SA concentration (Zhang *et al.*, 2003; Rochon *et al.*, 2006). However, upon pathogen-induced SA accumulation, NPR1 interacts with TGA2 and forms an enhancesome that activates *PR1* transcription (Boyle *et al.*, 2009). In the transcriptionally active NPR1–TGA2 complex, the N-terminal BTB domain of NPR1 interacts with and negates the function of the TGA2 repression domain (Boyle *et al.*, 2009). In addition, the oxidation of Cys521 and Cys529 in the C-terminal transactivation domain of NPR1 is required for the NPR1–TGA2 enhancesome (Rochon *et al.*, 2006).

Here, we report that a putative copper chaperone is an SA-responsive gene and induced by infections with plant pathogens, including plant viruses and bacteria. Unlike previously identified plant copper chaperones located in the cytoplasm, plasmids, and mitochondria, CCP harbors a nuclear localization signal (NLS) at its C-terminus that mediates its nuclear translocation. We further found that CCP could interact with TGA2 *in vivo* and *in vitro*, thereby inducing SA-mediated defense signaling.

## Materials and methods

### Construction of plasmids

The CCP ORF was inserted into the pMD19-T vector to generate pMD-CCP, which served as a template to generate pMD-CCP^mCBS^ (amino acid changes C13G/C16G) and pMD-CCP^mNLS^ (K61E, K62E, and F65E) using the QuikChange II kit (Stratagene, La Jolla, CA, USA). To generate pMDC32-CCP-Flag, pMDC32-CCP^mCBS^-Flag, pMDC32-CCP^mNLS^-Flag, and pMDC32-TGA2-Flag for transgenic or co-immunoprecipition assays, the CCP, CCP^mCBS^, CCP^mNLS^, and TGA2 cDNAs were fused to the N-terminus of three glycine residues and the Flag tag (3× Flag) of the binary vector pMDC32-3×Flag, a modified version of pMDC32 (Curtis and Grossniklaus, 2003).

To determine the expression pattern of the *CCP* gene, the genomic region from 2.5 kb upstream of the start codon to the stop codon of the *CCP* gene was engineered into the pBI101 vector (Clontech). For subcellular localization, the CCP ORF and derivatives were amplified from pMD19T-CCP and derivatives, and then inserted into pGDG (Goodin *et al.*, 2002). To generate pGFP-GUS-CCP and mutants, the β-glucuronidase (GUS) ORF was amplified from the pBI101 plasmid and was introduced in-frame to the position between green fluorescent protein (GFP) and CCP.

To construct *Arabidopsis thaliana ccp* mutant lines, two 19 bp sequences (see Supplementary Table S1 at *JXB* online) specifically targeting the 5' coding region of CCP were cloned into the pHEC401 vector as described previously (Xing *et al.*, 2014; Zhang *et al.*, 2019). All primers used for plasmid constructions are listed in Supplementary Table S1, and all the plasmids were verified by sequencing.

### Plant materials and growth conditions

For transient expression assays, H2B–red fluorescent protein (RFP) transgenic (Martin *et al.*, 2009) or wild-type *Nicotiana benthamiana* plants were used for agroinfiltration as described previously (Fang *et al.*, 2019). *Arabidopsis thaliana* seeds were incubated in 10% bleach for 15 min and washed with sterilized water five times, and then were plated on Murashige and Skoog (MS) medium. After vernalization at 4 °C for 3 d, plates were transferred into chambers with the condition of 23 °C and a 16 h light/8 h dark period for 10 d. Seedlings were then transferred into soil and grown in a room at 23 °C with a 10 h light/14 h dark cycle.

### Bacterial and viral inoculation assay

For bacterial infection assay, two fully grown leaves of 4-week-old Arabidopsis plants were infiltrated with *Pseudomonas syringae* pv. *tomato* DC3000 (*Pst* DC3000) strains as described previously (Li *et al.*, 2019). *Pst* DC3000 was cultured in King’s B liquid medium, washed in 10 mM MgCl_2_ twice, and diluted to OD_600_=0.0002 for infiltration. Infiltrated leaves were harvested for bacterial growth detection using colony-forming units per cm^2^. Bacterial growth was assessed in three independent experiments. For virus infection assay, three fully grown leaves of 4-week-old Arabidopsis plants were mechanically inoculated with *Cucumber mosaic virus* (CMV; 20 ng μl^–1^) as described previously (Zhang *et al.*, 2019). Systemically infected leaves were collected for RNA analyses.

### GUS staining

GUS staining assays were performed as described previously (Guo *et al.*, 2018). Briefly, plant tissues were treated with X-Gluc solution (3 mM 5-bromo-4-chloro-3-indolyl-β-d-glucuronide, 10 mM potassium ferricyanide, 0.1% Triton X-100, 10 mM potassium ferrocyanide, 50 mM NaH_2_PO_4_ buffer, pH 7.0) at 37 °C overnight, washed in an ethanol series, and photographed using a digital camera.

### Real-time quantitative reverse transcription–PCR (RT–PCR)

Total RNAs were extracted from *A. thaliana* tissues using TRIzol reagent following the manufacturer’s instructions (Invitrogen). Total RNA was treated with RNase-free DNase I (Takara) and served as template for reverse transcription using the M-MLV reverse transcriptase (Promega). Real-time quantitative PCRs were performed to examine accumulation of *CCP* and *PR1* mRNA with SsoFast EvaGreen Supermix (Bio-Rad). The *ACTIN2* gene served as an internal control. Specific primers are listed in Supplementary Table S1.

### Identification of terminal sequences of the *CCP* cDNA

3' and 5' RACE were performed as described previously (Yan *et al.*, 2015). Briefly, RNA samples were isolated from SA-treated *A. thaliana* plants, enriched by oligo(dT) beads, and used to identify two terminal sequences of the *CCP* mRNA with a SMARTer RACE 5′/3' Kit (Clontech, https://www.takarabio.com/). Amplified PCR products were cloned into pMD-19T for sequencing. Gene-specific primers for 5′/3' RACE are listed in Supplementary Table S1.

### Protein subcellular localization

For subcellular localization, the CCP cDNA and derivatives were cloned into pGDG (Goodin *et al.*, 2002), and the resulting plasmids were agroinfiltrated into H2B transgenic *N. benthamiana* leaves. At 2 days post-inoculaton (dpi), localization images were taken with a confocal laser scanning microscope (Zeiss LSM710). In addition, pGDG-CCP and derivatives were transformed into Arabidopsis protoplasts as described previously (Yoo *et al.*, 2007). The transformed Arabidopsis protoplasts were incubated at 20 °С for 18 h and were visualized by a Zeiss LSM710.

### Protein interaction assays

Bimolecular fluorescence complementation (BiFC) assays were performed to examine the interaction of CCP and TGA2 as described previously (Zhang *et al.*, 2017). CCP, TGA2 cDNA fragments, their derivatives, and the *Rubisco* gene (Rub) were introduced into the BiFC vectors pSPYNE-35S or pSPYCE-35S (Walter *et al.*, 2004). The recombinant BiFC plasmids and the *Tomato bushy stunt virus* P19 plasmid were transformed into *A. tumefaciens* EHA105 strains and co-infiltrated into *N. benthamiana* leaves. At 2 dpi, epidermal cells of infiltrated patches were monitored for yellow fluorescent protein (YFP) fluorescence by a Zeiss LSM710 confocal microscope (Carl Zeiss).

*In vivo* co-immunoprecipitation (Co-IP) of CCP and TGA2 was performed as described previously (Zhang *et al.*, 2017). TGA2-Flag was co-expressed with GFP, CCP–GFP, or CCP^mCBS^–GFP in *N. benthamiana* leaves through agroinfitration-mediated transient expression assays. Co-infiltrated leaf tissues were homogenized in Co-IP buffer [0.1% Triton X-100, 10% glycerol, 25 mM Tris–HCl (pH 7.5), 2% PVP-40, 200 mM NaCl, 1 mM EDTA, 10 mM DTT, and protease inhibitor cocktail]. After centrifugation, the resulting supernatants were mixed with anti-Flag M2 affinity gel (Sigma, https://www.sigmaaldrich.com/) at 4 °C for 5 h. The immunoprecipitated products were washed five times and boiled in SDS buffer for protein detection with corresponding antibodies.

For pull-down assays, TGA2 and GFP cDNAs were cloned into pGEX-4T-1, and the resulting recombinant plasmids were introduced into *Escherichia coli* (BL21 strain) for expression of GST–TGA2 and GST–GFP proteins, respectively. CCP and CCP^mCBS^ cDNAs were engineered into pET30a for expression of His-CCP and His-CCP^mCBS^, respectively. Glutathione *S*-transferase (GST)–TGA2, GST–GFP, His-CCP, and His-CCP^mCBS^ were expressed and purified according to the manufacturer’s instruction. For *in vitro* binding assays, 2 μg of bait proteins (GST–TGA2 or GST–GFP) and 2 μg of prey proteins (6× His-CCP and mutant) were incubated with GST beads in 500 μl of binding buffer [50 mM Tris–HCl (pH 6.8), 150 mM NaCl, 0.2% glycerol, 5 mM DTT, protease inhibitor cocktail, and 0.6% Triton X-100] at 4 °С for 6 h. After washing six times with binding buffer, the pull-down proteins were analyzed by immunoblotting using anti-His and GST antibody, respectively.

### EMSAs

For EMSAs, CCP and NPR1 were cloned into pGEX-4T-1 and the resulting recombinant plasmids were introduced into *E. coli* (BL21 strain) for expression of GST–CCP and GST–NPR1 proteins, respectively. EMSAs were performed using the LightShift Chemiluminescent EMSA Kit following the manufacturer’s instructions (Thermo Scientific, https://www.thermofisher.com/). Briefly, the *LS7* probe (5'-TATTTTACTTACGTCATAGATGTGGCGGCA-3' annealed to 5'-TGCCGCCACATCTATGACGTAAGTAAAATA-3') was labeled with biotin. A total of 500 ng of recombinant GST-tagged proteins were mixed with 4 fmol of biotin-labeled *LS7* probe in 20 μl reaction mixtures containing 1 μl of dI–dC, 1 μl of 50% glycerol, and 10× binding buffer for 20 min at 25 °С, and then separated on 6% native polyacrylamide gels. The labeled probes were detected according to the EMSA kit instructions.

### Yeast complementation

The yeast complementation assays were performed as described previously (Abdel-Ghany *et al.*, 2005). Briefly, the *Saccharomyces cerevisiae lys7* mutant was generated by replacing the *Lys7* gene with the *LEU2* gene in the yeast BY4741 strain. The CCP cDNA and derivatives were cloned into pGPD-416-1 and the resulting plasmids were transformed into the *lys7* mutant. The SC minimal medium lacking leucine and uracil was used to select positive transformed colonies. For complementation assays, serial dilutions of transformed yeasts were plated on SC minimal medium lacking leucine and uracil with or without menadione (Sigma) at 30 °C for 3 d.

### ChIP assays

ChIP assays were performed as described previously (Wang *et al.*, 2019). Briefly, Arabidopsis transgenic lines CCP^OE^ and CCP^mCBS/OE^ were grown in a room at 23 °C with a 10 h light/14 h dark cycle for ChIP. Fully expanded leaves of 4-week-old seedlings were inoculated with *Pst* DC3000 and harvested at 1 dpi for ChIP assays. The inoculated leaves (2 g) were treated with 1% formaldehyde to cross-link the protein–DNA complexes under vacuum. Then, the samples were isolated, sonicated, and centrifuged at 12 000 *g* at 4 °C for 10 min. The supernatant was transferred into new tubes and incubated with anti-Flag affinity beads. The precipitated DNA samples were quantified by quantitative PCR using the primers listed in Supplementary Table S1.

## Results

### Identification of a putative copper chaperone induced by plant pathogens

**Fig. 1.**
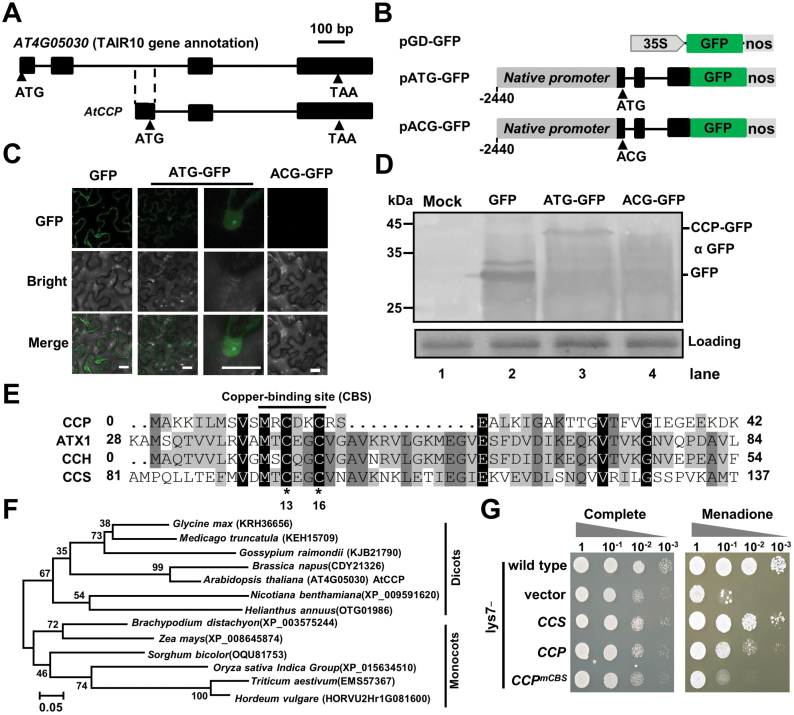
Identification of a novel copper chaperone in Arabidopsis. (A) Genome structure of *AT4G05030* from TAIR10 gene annotation (upper) or the identified *CCP* cDNA (bottom). Exons and introns are indicated by black boxes and lines, respectively. The start codon (ATG) and stop codon (TAA) of the predicted small ORF are indicated by arrowheads. (B) Illustration of binary vectors for expressing GFP or GFP-fused proteins in *N. benthamiana* leaves. In pATG-GFP, the DNA genome fragment between 2440 bp upstream of the CCP start codon to the end of the CCP ORF was amplified from Col-0 and fused to the N-terminus of GFP. In pACG-GFP, the predicted start codon ATG of pATG-GFP was mutated to ACG. (C) Confocal micrographs examining GFP, ATG–GFP, or ACG–GFP expression in agroinfiltrated *N. benthamiana* leaves at 3 dpi. Scale bar=20 μm. (D) Western blotting analyses showing accumulation of GFP, ATG–GFP, or ACG–GFP in the samples of (C) with anti-GFP antibody. Buffer-infiltrated leaves served as a mock control. The Coomassie brilliant blue staining was used as the protein loading control. (E) Alignments of Arabidopsis CCP, ATX1, CCH, and CCS. The conserved copper-binding site (CBS) is indicated by a line. (F) Phylogenetic trees of plant CCP orthologs based on amino acid sequences. Accession numbers or locus are shown. Scale bar=0.05 amino acid substitutions per site. (G) Complementation of the growth phenotype of the *S. cerevisiae lys7* mutant cells transformed with empty plasmid (vector), or plasmids expressing Arabidopsis CCS, CCP, or CCP^mCBS^. Wild-type *S. cerevisiae* and complemented *lys7* mutant cells were assayed for growth on complete medium or medium with 25 μM menadione. Cells were spotted in 10-fold serial dilutions starting at OD_600_=0.1, and incubated at 30 °C for 3 d. (This figure is available in color at *JXB* online.)

To investigate host transcriptome response to virus infection, total RNA from CMV-infected *A. thaliana* leaves or mock-treated leaves was used for transcriptome analysis. Among up-regulated genes, the *AT4G05030* mRNA accumulated to a low level in mock-treated leaves, but was induced by CMV infection (Supplementary Fig. S1A). Moreover, 5′ and 3' RACE assays revealed that the *AT4G05030* cDNA *in vivo* corresponds to part of the second intron, and third and fourth exons of the TAIR10 gene annotated as *AT4G05030* cDNA (Fig. 1A; Supplementary Fig. S1B).

Interestingly, a small ORF was predicted from the identified *AT4G05030* cDNA (Fig. 1A; Supplementary Fig. S1C). To confirm the ORF translation, the *AT4G05030* genomic region from 2440 bp upstream of the start codon to the end of the ORF was fused to the GFP N-terminus to generate pATG-GFP (Fig. 1B). Moreover, the pACG-GFP mutant was generated by replace the new start codon ATG with ACG (Fig. 1B). *Agrobacterium* harboring the pGD-GFP, pATG-GFP, or pACG-GFP plasmids was agroinfiltrated into *N. benthamiana* leaves. At 5 dpi, GFP fluorescent speckles were observed in the nuclei of leaf epidermal cells infiltrated with pATG-GFP, but not in those infiltrated with pACG-GFP (Fig. 1C). Western blotting analyses revealed that the ATG–GFP protein was larger than GFP protein (Fig. 1D), indicating that the *AT4G05030* small ORF was translated *in vivo*.

The small ORF of *AT4G05030* encodes a 77 amino acid polypeptide harboring a classical copper-binding motif, MXCXXC, that is conserved in well-studied copper chaperones, such as ATX1, CCH, and CCS (Fig. 1E). Thus, *AT4G05030* probably encodes a putative CCP. Blast searches reveal that many monocot and dicot plant species harbor a single CCP ortholog (Fig. 1F), implying a conserved function of CCP orthologs in plants. However, all CCP orthologs have not been characterized previously.

To investigate whether CCP is a functional homolog of the yeast copper chaperone Ccs1/Lys7, Arabidopsis CCS and CCP were expressed in the yeast *lys7* mutant (Supplementary Fig. S2). The functional complementation assay was performed by analyzing the growth phenotype on complete medium or medium containing the superoxide generator menadione. As expected, ectopic expression of Arabidopsis CCS completely rescued SOD activity of the *lys7* mutant, whereas expression of CCP partially rescued SOD activity (Fig. 1G). In contrast, ectopic expression of the CCP^mCBS^ mutant harboring substitution of the conserved Cys13 and Cys16 with glycine could not rescue the SOD activity in the *lys7* mutant (Fig. 1G). These results suggest that CCP can partially protect the *lys7* mutant from reactive oxygen toxicity in a copper binding-dependent manner.

### The nuclear translocation of CCP requires the CBS and NLS motifs

**Fig. 2.**
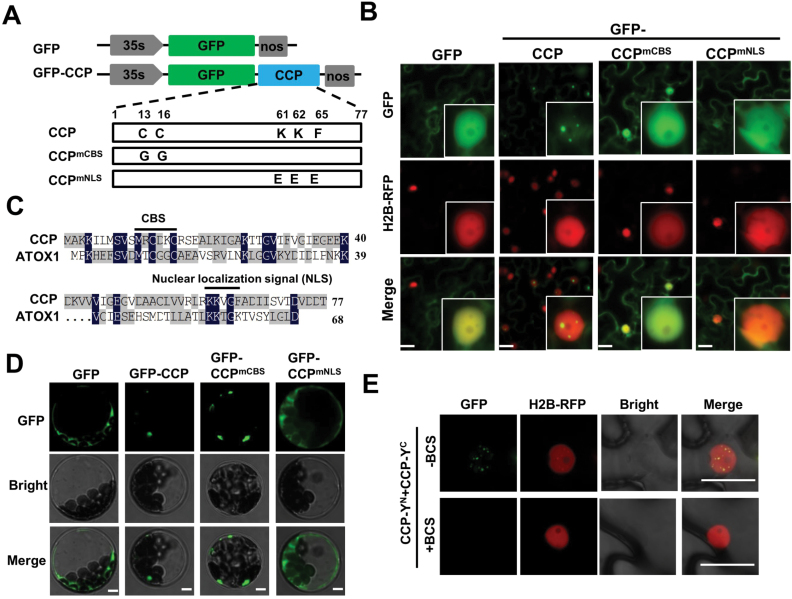
Subcellular localization of CCP and derivative mutants. (A) Diagram illustrating GFP–CCP, GFP–CCP^mCBS^, and GFP-CCP^mNLS^. Mutated amino acids are indicated in boxes. (B) Confocal micrographs showing the subcellular localization of GFP, GFP–CCP, GFP–CCP^mCBS^, and GFP–CCP^mNLS^ in agroinfiltrated leaves of transgenic *N. benthamiana* plants with H2B–RFP, a nuclear marker. Photographs were taken at 3 dpi. Scale bar=20 μm. (C) Alignment of CCP and mammalian ATOX1. A copper-binding site (CBS) and a predicted nuclear localization signal (NLS) are indicated by lines. (D) Fluorescence micrographs showing subcellular localization of GFP (control), GFP–CCP, GFP–CCP^mCBS^, and GFP–CCP^mNLS^ in Arabidopsis protoplasts. Scale bar=5 μm. (E) BiFC analyzing the CCP self-interaction in epidermal cells of *N. benthamiana* leaves treated with buffer or BCS, a copper chelator. Scale bar=5 μm. (This figure is available in color at *JXB* online.)

To determine CCP subcellular localization, *Agrobacterium* harboring plasmids for expression of GFP–CCP or GFP (Fig. 2A) were infiltrated into leaves of H2B–RFP transgenic *N. benthamiana* plants (Martin *et al.*, 2009). At 3 dpi, confocal examinations revealed that free GFP localized evenly in the cytoplasm and nucleus (Fig. 2B; Supplementary Fig. S3A). In contrast, GFP–CCP predominantly formed fluorescent nuclear speckles in the nucleus (Fig. 2B; Supplementary Fig. S3A). The mammalian antioxidant-1 (ATOX1) protein has 68 amino acids and an NLS at the C-terminus (Fig. 2C) (Itoh *et al.*, 2008; Muller ansd Klomp, 2009). Amino acid sequence alignment revealed that a KKVGF motif at the CCP C-terminus might be a potential NLS (Fig. 2C).

To determine the requirement for the CBS and NLS motifs for subcellular localization, GFP–CCP^mCBS^ and GFP–CCP^mNLS^ were further expressed in H2B–RFP transgenic *N. benthamiana* plants (Fig. 2A). Western blotting revealed expression of GFP, GPF–CCP, GFP–CCP^mCBS^, and GFP–CCP^mNLS^ in infiltrated leaves (Supplementary Fig. S3). Confocal examination revealed that GFP–CCP^mCBS^ and GFP–CCP^mNLS^ localized evenly in the cytoplasm and nucleus, which is distinct from the nuclear speckles of GFP–CCP (Fig. 2B). Consistently, GFP–CCP fluorescence was observed mainly in the nuclei of transfected Arabidopsis protoplasts, whereas GFP, GFP–CCP^mCBS^, and GFP–CCP^mNLS^ fluorescence was observed in the cytoplasm of transfected protoplasts (Fig. 2D). To rule out non-specific diffusion of the small GFP–CCP protein into the nucleus, CCP, CCP^mCBS^, and CCP^mNLS^ were fused with GFP–GUS and expressed in H2B–RFP transgenic *N. benthamiana* leaves. As expected, GFP fluorescence from GFP–GUS–CCP was predominantly present in the nuclei (Supplementary Fig. S4), whereas GFP–GUS–CCP^mCBS^ and GFP–GUS–CCP^mNLS^ were limited in the cytoplasm (Supplementary Fig. S4). These results clearly indicate that both the CBS and NLS motifs are required for CCP nuclear localization.

To explore whether self-interactions of CCP were required for CCP nuclear localization, CCP, CCP^mNLS^, and CCP^mCBS^ were fused to the N (Y^N^) or C (Y^C^) halves of sYFP, and then co-expressed in *N. benthamiana* leaves. BiFC assays revealed that self-interactions of CCP and CCP^mNLS^ were detected in the nuclei and cytoplasm, respectively (Supplementary Fig. S5A). In contrast, self-interaction of CCP^mCBS^ was not detected although all the proteins were expressed (Supplementary Fig. S5A, C). The Y^N^- or Y^C^-fused transcription factor ELONGATED HYPOCOTYL 5 (HY5) (Oyama *et al.*, 1997) served as a negative control that did not exhibit BiFC fluorescence with other combinations (Supplementary Fig. S5C). We further treated the CCP-Y^N^/CCP-Y^C^ co-infiltrated leaves with buffer or the copper chelator bathocuproine sulfonate (BCS) (Itoh *et al.*, 2008). The CCP-Y^N^/CCP-Y^C^ interaction in the nucleus was nearly abolished in BCS-treated leaves compared with the control treatment (Fig. 2E). In addition, the GFP–CCP-expressing leaves were further treated with mock buffer or BCS, showing that the cytoplasmic localization of GFP–CCP increased compared with the mock control (Supplementary Fig. S6). Collectively, these results indicate that CBS-dependent self-interaction is required for CCP nuclear localization.

Overall, these results indicate that both the CBS and NLS motifs are required for CCP nuclear localization. The *N. benthamiana* CCP ortholog (NbCCP) had similar nuclear translocation and an NLS (Supplementary Fig. S7A). However, Arabidopsis ATX1 and CCH did not localize in the plant nucleus (Supplementary Fig. S7B), which was in agreement with previous studies (Puig *et al.*, 2007). To ensure non-specific diffusion of the small GFP–ATX1/CCH protein into the nucleus, GFP–GUS–ATX1 and GFP–GUS–CCH were expressed in H2B–RFP transgenic *N. benthamiana* leaves, showing that these fusion proteins were exclusively in the cytoplasm (Supplementary Fig. S7B). Western blotting revealed expression of GFP–ATX1, CCH, GUS–ATX1, and GUS–CCH in H2B–RFP transgenic *N. benthamiana* leaves (Supplementary Fig. S7C). Thus, the nuclear localization of CCP is a unique feature among the identified plant copper chaperones.

### *CCP* is induced by plant pathogen infections and SA treatment

**Fig. 3.**
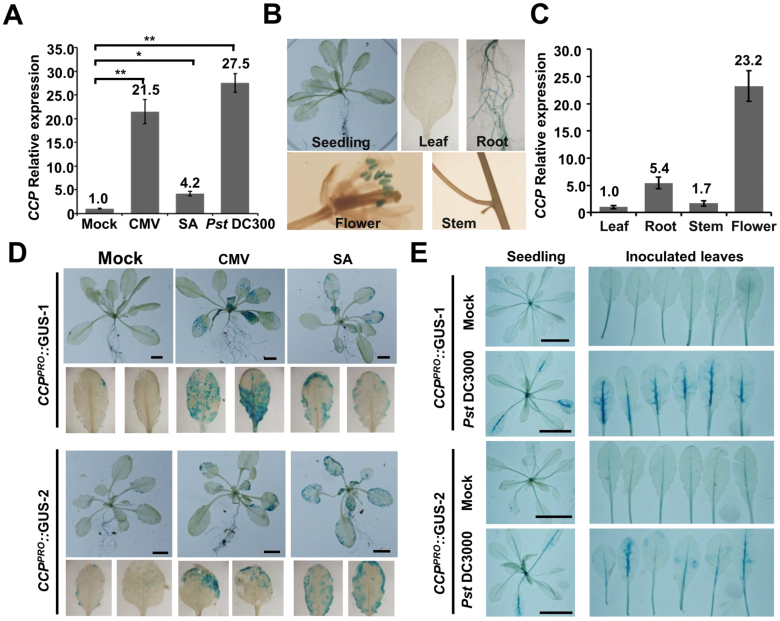
The tissue expression pattern of Arabidopsis *CCP*. (A) Quantitative RT–PCR analyzing *CCP* mRNA accumulation in systemic leaves of CMV-infected plants at 7 dpi, SA-treated leaves at 3 dpi, and *Pst* DC300-inoculated leaves at 1 dpi. Accumulation of *CCP* mRNA in mock-treated leaves was set as one unit. Numbers above columns are mean values of three independent experiments. Error bars represent the SEM. *P-value <0.05; **P-value <0.01. (B) Histochemical GUS activities in different tissues of *CCP*^*PRO*^*::*GUS transgenic plants. (C) Relative accumulation of the *CCP* mRNA in different tissues of Col-0 plants. Accumulation of *CCP* mRNA in leaves was set as one unit. Numbers above columns are mean values of three independent experiments. Error bars represent the SEM of three independent experiments. (D) GUS activity in two independent *CCP*^*PRO*^*::*GUS transgenic lines treated with buffer, CMV infection, or SA. Detached systemically infected leaves are shown in the bottom panels. Scale bar=5 cm. (E) GUS activity in *CCP*^*PRO*^*::*GUS-1 and *CCP*^*PRO*^*::*GUS-2 after mock treatment or *Pst* DC3000 infection at 1 dpi. Detached inoculated leaves are shown in the right-hand panels. Scale bar=3 cm. (This figure is available in color at *JXB* online.)

To determine *CCP* response to pathogen infections, Col-0 leaves were treated by mock buffer, CMV, *Pst* DC3000, or SA, and then collected for quantitative RT–PCR. Compared with mock treatment, CMV, SA, and *Pst* DC3000 treatment induced *CCP* accumulation by ~21.5-, 4.2-, and 27.5-fold (Fig. 3A). Furthermore, transgenic Col-0 plants expressing GUS under control of the *CCP* native promoter were generated to investigate the *CCP* tissue expression pattern. GUS staining results revealed that GUS activity was detected at higher levels in roots and flowers than in leaves and stems of healthy *CCP*^*PRO*^*::*GUS transgenic plants (Fig. 3B), which was further verified by quantitative RT–PCR assays (Fig. 3C). Two independent *CCP*^*PRO*^*::*GUS transgenic lines were infected with CMV or treated with SA, revealing that GUS expression was induced by CMV infection and SA treatment (Fig. 3D). Similarly, *Pst* DC3000 inoculation resulted in increased GUS activity in infiltrated leaves, especially in the veins (Fig. 3E). However, we found that the *CCP* gene was suppressed but not induced by excess copper (Supplementary Fig. S8), which is consistent with the *CCH* gene (Shin *et al.*, 2012). Collectively, our findings demonstrate that *CCP* is transcriptionally induced by pathogen infections and SA treatment.

### CCP enhances plant resistance to bacterial pathogens

We next examined whether CCP is involved in resistance against bacterial pathogens. To this end, we generated CCP-overexpressing transgenic lines (CCP^OE^) under control of the *Cauliflower mosaic virus* (CaMV) 35S promoter, in which increased accumulation of *CCP* was verified by RT–PCR (Supplementary Fig. S9A). Accumulation of the CCP protein in CCP^OE^-3 and CCP^OE^-4 was confirmed by western blotting analyses for functional experiments (Supplementary Fig. S9B). In addition, we generated two *ccp* mutants using the CRISPR/Cas9 [clustered regularly interspaced palindromic repeats (CRISPR)/CRISPR-associated protein 9] system with the guide RNA targeting the downstream region of the start codon (Supplementary Fig. S10). The resulting *ccp-1* and *ccp-2* contain a 445 bp deletion and a 1 bp insertion, respectively (Supplementary Fig. S10).

**Fig. 4.**
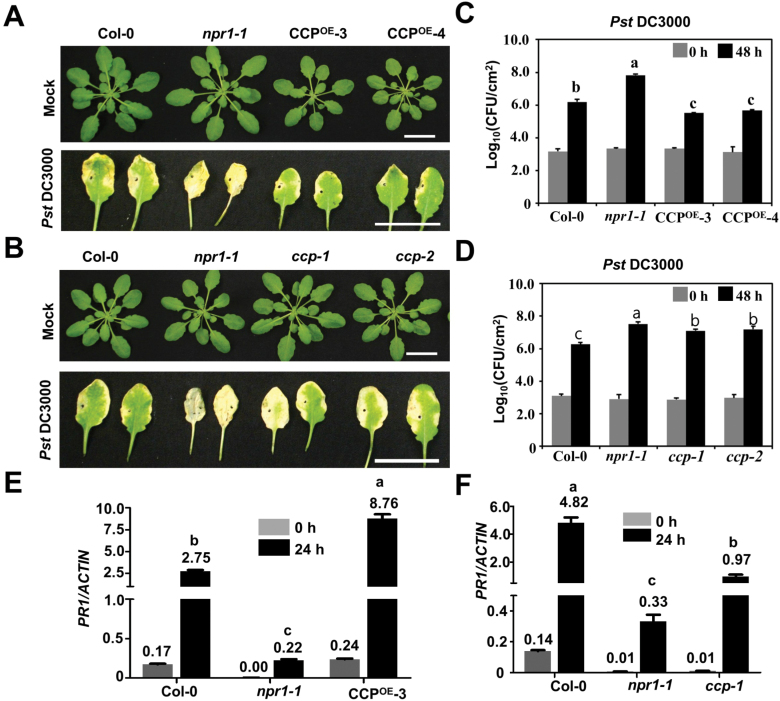
CCP improves plant resistance against bacterial pathogens. (A) Morphology and symptom development of wild-type Col-0, *npr1-1*, CCP^OE^-3-, and CCP^OE^-4 leaves inoculated with buffer or *Pst* DC3000 at 72 hpi. Scale bar=2 cm. (B) Morphology and symptom development of wild-type Col-0, *npr1-1*, *ccp-1*, and *ccp-2* leaves inoculated with buffer or *Pst* DC3000 at 72 hpi. Scale bar=2 cm. (C) Growth of *Pst* DC3000 on infiltrated leaves of Col-0, *npr1-1*, CCP^OE^-3, and CCP^OE^-4 plants at 0 and 48 hpi. Four-week-old plants were infiltrated with *Pst* DC3000 (OD_600_=0.0002). CFU, colony-forming units. (D) Growth of *Pst* DC3000 on infiltrated leaves of Col-0, *npr1-1*, *ccp-1*, and *ccp-2* plants at 0 and 48 hpi. (E and F) Relative ratio of the PR1 mRNA versus the endogenous actin mRNA in Col-0, *npr1-1*, CCP^OE^-3 (E), or *ccp-1* (F) leaves infiltrated with *Pst* DC3000 (OD_600_=0.0002) at 0 and 24 hpi. In (C–F), scale bars represent means ±SE (*n*=3). Different letters indicate significant differences (*P*<0.05, *n*=3) determined by ANOVA by Turkey’s multiple comparison test analysis. (This figure is available in color at *JXB* online.)

Rosette leaves of 4-week-old Col-0, CCP^OE^-3, CCP^OE^-4, *ccp-1*, *ccp-2*, and *npr1-1* were infiltrated with *Pst* DC3000. At 0 and 48 h post-inoculation (hpi), infiltrated leaves were collected for assessing bacterial growth by counting colony-forming units. The *npr1-1* mutant allowed much higher accumulation of *Pst* DC3000 at 48 hpi and exhibited enhanced chlorotic symptoms at 72 hpi compared with Col-0 leaves (Fig. 4A–C). The infiltrated leaves of CCP^OE^-3 and CCP^OE^-4 exhibited milder symptoms (Fig. 4A), whereas more severe chlorotic symptoms were observed in the *ccp-1* and *ccp-2* mutant leaves (Fig. 4B). Consistently, CCP^OE^-3 and CCP^OE^-4 allowed a lower level of *Pst* DC3000, while the *ccp-1* and *ccp-2* mutants supported an increased level of bacteria compared with Col-0 plants (Fig. 4C, D). Consistently, compared with the infected Col-0 leaves, *PR1* mRNA accumulated to a substantially higher level in CCP^OE^-3 leaves (Fig. 4E) and a reduced level in *ccp-1* leaves (Fig. 4F). Collectively, CCP enhances plant defense against *Pst* DC3000.

### Interaction of CCP with the N-terminal repression domain of TGA2

**Fig. 5.**
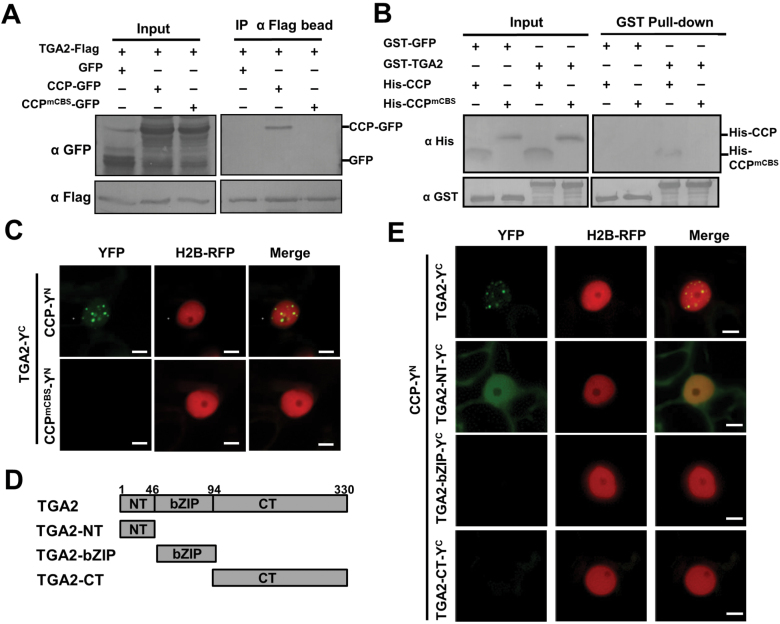
Interaction of CCP with TGA2 *in vivo* and *in vitro*. (A) Co-IP assays showing the interaction of TGA2 and CCP *in vivo*. *Nicotiana benthamiana* leaves were agroinfiltrated with constructs for co-expressing TGA2-Flag with CCP–GFP, CCP^mCBS^–GFP, or GFP. At 2 dpi, total proteins isolated from the agroinfiltrated *N. benthamiana* leaves were immunoprecipitated with anti-Flag beads. Total proteins (Input) and immunoprecipitated proteins (IP) were analyzed by western blotting analyses with anti-Flag and anti-GFP antibodies. (B) GST pull-down analysis of the interaction between TGA2 and CCP *in vitro*. GST–GFP or GST–TGA2 were incubated with His-CCP or His-CCP^mCBS^, and then immunoprecipitated with glutathione–Sepharose beads *in vitro*. The input and pull-down products were detected by western blotting analysis with anti-GST or anti-His antibodies. (C) BiFC analysis of the interaction of TGA2 with CCP or CCP^mCBS^ in H2B transgenic *N. benthamiana* leaves. *Agrobacterium* strains harboring plasmids expressing Y^N^- or Y^C^-tagged tested proteins were infiltrated in H2B transgenic *N. benthamiana* leaves. At 2 dpi, BiFC fluorescence was monitored by confocal scanning laser microscopy. Scale bar=5 μm. (D) Schematic diagrams of the TGA2 domains. (E) BiFC analysis of the interaction between the three main domains of TGA2 and CCP in H2B transgenic *N. benthamiana* leaves. Scale bar=5 μm. (This figure is available in color at *JXB* online.)

Upon plant pathogen infections and SA treatment*, CCP* expression is induced and undergoes nuclear translocation mediated by CBS and NLS motifs. Moreover, CCP is required for plant resistance to bacterial pathogens. Given that the TGA transcription factors are important regulators in the SA signaling pathway, we hypothesized that TGA transcription factors might be targeted by CCP in the nucleus. To verify this hypothesis, we performed Co-IP assays to examine the potential interaction of CCP and TGA2. The TGA2-Flag protein was co-expressed with CCP–GFP or CCP^mCBS^–GFP in *N. benthamiana* leaves by agroinfiltration. At 2 dpi, the TGA2-Flag protein and associated proteins were precipitated with anti-Flag affinity beads. Western blotting analyses revealed that TGA2-Flag was precipitated with CCP–GFP, but not with CCP^mCBS^–GFP or free GFP (Fig. 5A). *In vitro* pull-down assays were further carried out to determine the direct interaction of CCP and TGA2 using CCP-His, CCP^mCBS^-His, GST–GFP, and GST–TGA2. Pull-down assays revealed that CCP-His, but not CCP^mCBS^-His, was pulled down with GST–TGA2 (Fig. 5B). On the other hand, the GST–GFP control did not interact with CCP-His or CCP^mCBS^-His (Fig. 5B).

We next determined whether CCP associates with TGA2 *in vivo* using a BiFC assay. To this end, the TGA2 coding sequence was fused with the C-terminal half of YFP (TGA2-Y^C^), and coding sequences of CCP or CCP^mCBS^ were fused with the N-terminal half of YFP (CCP-Y^N^ or CCP^mCBS^-Y^N^). Co-expression of TGA2-Y^C^ and CCP-Y^N^ resulted in YFP fluorescent speckles in the nuclei of infiltrated cells (Fig. 5C). Interestingly, the SA treatment increase the YFP intensity of TGA2-Y^C^ and CCP-Y^N^ in the nucleus compared with mock treatment (Supplementary Fig. S11). In contrast, leaf cells co-expressing TGA2-Y^C^ with CCP^mCBS^-Y^N^ or negative control HY5-Y^N^ failed to produce YFP signal (Fig. 5C; Supplementary Fig. S5B).

TGA2 contains an N-terminal domain (amino acids 1–46), a middle bZIP domain (amino acids 47–94), and a C-terminal domain (amino acids 95–330) (Fig. 5D) (Rochon *et al.*, 2006; Boyle *et al.*, 2009). To determine the CCP-binding domain in the TGA2 protein, the three domains were individually fused with Y^C^ and co-expressed with CCP-Y^N^ in *N. benthamiana* leaves. BiFC results revealed that the NT domain of TGA2 interacted with the CCP protein *in vivo* (Fig. 5E). It should be noted that the BiFC signal of CCP-Y^N^ and TGA2-NT-Y^C^ localized evenly in the cytoplasm and nucleus, which was different from the speckles formed by CCP-Y^N^ and TGA2-Y^C^ (Fig. 5E). To explain this different localization, GFP–TGA2, GFP–NT, GFP–bZIP, and GFP–CT were expressed in H2B–RFP transgenic *N. benthamiana* plants through agroinfiltration. The results showed that GFP signals were observed in speckles mainly in the nuclei as GFP–TGA2, whereas GFP signals from GFP–NT and GFP–CT localized evenly in the nuclei and cytoplasm. On the other hand, GFP signals from GFP–bZIP were mainly present in the nucleolus (Supplementary Fig. S12). Collectively, these results indicate that CCP interacts directly with TGA2, and that the CCP CBS motif and the TGA2 NT domain are essential for their interactions.

### CCP functions together with TGA2 to activate the expression of *PR1*

**Fig. 6.**
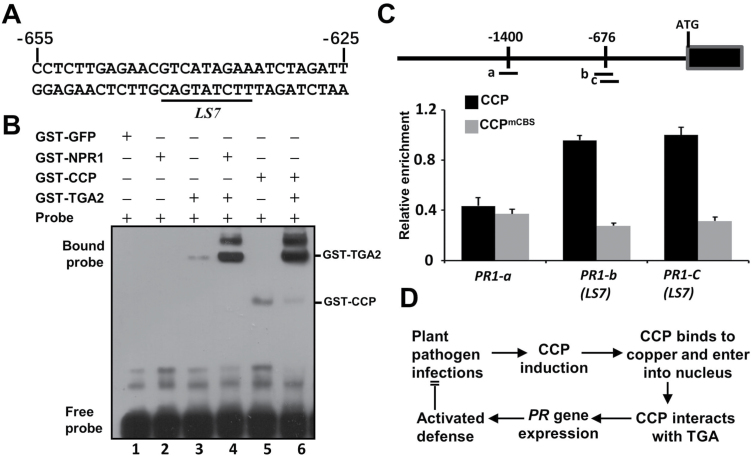
CCP enhances the binding activity of TGA2 to the LS7 element of the *PR1* promoter *in vivo*. (A) The nucleotide sequence of the LS7 element (underlined) and surrounding region in the *PR1* promoter. The numbers on the sequence indicate the position of the nucleotide relative to the transcription start site of the PR1 mRNA. The sequence was used as a probe in EMSAs. (B) EMSA showing the binding of TGA2 with the LS7 element of the *PR1* promoter. GST-tagged GFP, TGA2, NPR1, and CCP proteins were purified from *E. coli* and mixed in different combinations. Equal amounts of the biotin-labeled LS7 element were incubated with different protein combinations, and the DNA binding was analyzed by EMSAs. (C) ChIP-qPCR assays showing binding of CCP to fragments of the *PR1* promoter *in vivo*. Chromatins from *35S:CCP* and *35S:CCP*^*mCBS*^ transgenic Arabidopsis seedlings with anti-Flag beads were collected for DNA extraction and quantitative PCR assays. Non-transgenic Col-0 plants acted as a negative control. Mean ±SD, *n*=3. Top, diagram of the *PR1* promoter and the fragments for Chip-qPCR. (D) A proposed working model of CCP-mediated defense activation. Upon plant pathogen challenges, CCP is induced and binds to copper. CCP is translocated into the nucleus and interacts with TGA2, resulting in induction of defense response genes and improved immunity against plant pathogens.

Previous studies have shown that TGA2 binds to the linker scan 7 element (*LS7*; Fig. 6A) of the *PR1* promoter to positively regulate *PR1* expression (Zhang *et al.*, 1999). The NPR1 protein enhances the DNA binding affinity of TGA2 and then activates defense gene expression (Després *et al.*, 2000). Since the N-terminus of TGA2 has been shown to be a repression domain (Boyle *et al.*, 2009), the interaction of CCP and the N-terminal domain of TGA2 may enhance the binding of TGA2 to the *LS7* element of the *PR1* promoter and then activates pathogenesis-related gene expression. To test the hypothesis, the *LS7* element and surrounding region of the *PR1* promoter was labeled by biotin for EMSAs (Fig. 6A). The *LS7* probe was incubated with different combinations of GST-tagged GFP, TGA2, NPR1, CCP, and TGA2. No retarded bands were observed on incubation of the *LS7* probe with GST–GFP or GST–NPR1 (Fig. 6B), indicating that GST, GFP, or NPR1 do not bind to the *LS7* probe. In contrast, incubation with GST–TGA2 or GST–CCP resulted in retarded bands of the *LS7* probe, indicating that TGA2 and CCP have faint binding activity of the *LS7* probe (Fig. 6B, lanes 3 and 5). Furthermore, GST–TGA2 incubated with GST–NPR1 or GST–CCP binds to a substantially increased amount of the *LS7* probe compared with GST–TGA2 alone (Fig. 6B, compare lanes 4 and 6 with 3). Therefore, these results suggest that CCP and NPR1 enhance the binding of TGA2 with the positive regulatory element (*LS7*) of the *PR1* promoter.

To examine whether CCP is associated with the *PR1* promoter *in vivo*, we performed ChIP-PCR assays using 4-week-old seedlings of Col-0, CCP^OE^-3, and CCP^mCBS/OE^ infected with *Pst* DC3000. CCP^mCBS/OE^-1 was generated by transforming Col-0 with CCP^mCBS^ under the control of the CaMV 35S promoter. The qRCR results revealed that ‘b’ and ‘c’ amplicons containing the *LS7* element of the *PR1* promoter were enriched in the CCP^OE^-3 immunoprecipitated samples compared with CCP^mCBS/OE^ (Fig. 6C). In contrast, the ‘a’ amplicon located 1400 bp upstream of the *PR1* start codon was not enriched in the CCP^OE^-3 samples (Fig. 6C). These results indicate that CCP is preferentially associated with the TGA2-targeting *LS7* element of the *PR1* promoter.

Collectively, CCP is induced by plant pathogen infections and SA treatment. The CBS and NLS motif mediate the nuclear translocation, and then physically bind TGA2 *in vivo* at the *LS7* element of the *PR1* promoter to induce expression of defense genes (Fig. 6D).

## Discussion

Copper is an essential nutrient in various biological processes, but highly concentrated free copper is extremely toxic to cells. Thus, intracellular copper must be chelated by copper chaperones to avoid copped-induced detrimental effects. In addition, copper chaperones are responsible for copper delivery to specific copper proteins and compartments. In previous studies, three copper chaperone classes were named based on their interaction proteins and functions: interaction with SODs (CCS), interaction with copper-transporting ATPases (ATX and CCH), and function in mitochondrial respiration (COX17, COX11, HCC1, and HCC2) (Burkhead *et al.*, 2009; Attallah *et al.*, 2011). Here, we have identified a novel class of putative copper chaperone (CCP) that was induced by pathogen infections and SA treatment. In healthy plants, CCP was mainly expressed in roots and inflorescence tissues, and rapidly up-regulated upon infections with CMV and *Pst* DC3000, as well as SA treatment (Fig. 3). These results suggest that CCP has important roles in defense responses to pathogen infections. Blast search revealed that many monocot and dicot plants have a single CCP ortholog (Fig. 1F), indicating that the conserved CCP orthologs provide a common strategy for regulation of defense response in plants.

In plants, the copper-dependent biological processes have been extensively elucidated in the cytoplasm (Pilon *et al.*, 2006; Robinson and Winge, 2010). However, whether copper is transferred into the nucleus and mediates plant defense responses has not yet been investigated (Pilon *et al.*, 2006; Robinson and Winge, 2010). Intriguingly, we found that CCP predominantly formed nuclear speckles (Fig. 2). The mammalian ATOX1 has been shown to be a copper-dependent transcription regulator in the nucleus (Itoh *et al.*, 2008; Muller and Klomp, 2009; Kamiya *et al.*, 2018). A lysine-rich region (KKTGK) in the C-terminus of ATOX1 is an NLS (Hamza *et al.*, 2001; Itoh *et al.*, 2008; Muller and Klomp, 2009). The alignment of CCP with ATOX1 revealed that a KKVGF at the C-terminus of CCP was an NLS, which was supported by point mutation analyses (Fig. 2). In contrast, ATX1 and CCH do not localize to the nucleus (Supplementary Fig. S12). Thus, CCP represents a putative plant copper chaperone functioning in the nucleus.

The mammalian ATOX1 protein requires copper for nuclear translocation and transcriptional activation in the nucleus (Itoh *et al.*, 2008; Muller and Klomp, 2009). Furthermore, copper-dependent dimerization of ATOX1 was revealed by X-ray crystallography and biochemical analyses (Wernimont *et al.*, 2000). Thus, it has been proposed that ATOX1 homodimerization might block interaction with its cytoplasmic interactors, ATP7A and ATP7B of P-type ATPases, and trigger ATOX1 to translocate into the nucleus to regulate transcription (Muller and Klomp, 2009). In agreement with mammalian ATOX1, plant CCP also requires copper binding for its nuclear translocation, self-interaction activity, and interaction with TGA2 (Figs 2, 6). In addition, it should be noted that CCP might also target some components for copper delivery and mediate unknown biological processes in the cytoplasm.

Mammalian ATOX1 has been shown to be a copper-dependent transcription regulator; however, details of the functions of ATOX1 in these processes remain elusive (Itoh *et al.*, 2008; Muller and Klomp, 2009; Kahra *et al.*, 2015; Kamiya *et al.*, 2018; Matson Dzebo *et al.*, 2018). Previous studies found that ATOX1 could directly bind the GAAAGA promoter sequence as a transcription factor (Itoh *et al.*, 2008; Muller and Klomp, 2009; Kamiya *et al.*, 2018). However, other studies found that ATOX1 had no DNA binding activities *in vitro*, and regulated gene transcription via DNA-binding transcription regulators. Here, CCP moves into the nucleus and activates expression of defense genes. We detected slight binding activity of CCP with the elements in the promoter of *PR1* (Fig. 6A). Moreover, CCP may regulate *PR1* gene expression by interacting with the TGA2 transcription factor that binds to the as-1 elements of the *PR* gene promoters (Zhang, 1999; Zhou, 2000; Johnson *et al.*, 2003). In normal conditions, TGA2 is a transcriptional repressor that requires its N-terminus as a repression domain (Zhang *et al.*, 2003; Rochon *et al.*, 2006; Boyle *et al.*, 2009). Upon pathogen infection or SA treatment, the N-terminal BTB/POZ domain of NPR1 interacts with TGA2, masking its repressor domain and forming an enhancesome with TGA2 to activate *PR1* transcription (Boyle *et al.*, 2009). Similarly, we found that CCP also interacted with the N-terminal repression domain of TGA2 (Fig. 5E), which induced PR1 expression probably by inactivating the repression activity of TGA2. In agreement with this hypothesis, the CCP protein can induce *PR1* expression and enhance immunity against *Pst* DC3000 (Fig. 4). Given that TGA2, TGA5, and TGA6 act as redundant transcription factors in the SA signaling pathway (Zhang *et al.*, 2003; Dröge-Laser *et al.*, 2018), whether CCP interacts with TGA5 and TGA6 needs to be established in future studies. More interestingly, we found that GST–NPR1, rather than the negative control GST–GFP, could pull-down His-CCP and His-TGA2 together in the presence of Cu^2+^ (Supplementary Fig. S13), indicating that NPR1, CCP, and TGA2 are probably in a complex.

Taken together, our results provide insights that CCP is a putative copper chaperone family inducing defense against plant pathogens in the nucleus. As summarized in Fig. 6D, upon pathogen infections, the up-regulated CCP forms copper-dependent homodimers, and then undergoes nuclear translocation. Within the nucleus, the CCP homodimer binds the N-terminus of TGA2 that has been shown to be a repressor of transcription of PR1 without SA induction (Zhang *et al.*, 2003; Mosher *et al.*, 2006; Rochon *et al.*, 2006; Boyle *et al.*, 2009). The CCP homodimer binds to and masks the N-terminal repressive domain of TGA2, which probably results in negating the repression activity of TGA2 and positively regulating defense response genes to improve immunity against plant pathogens. There are still some difference between CCP and NPR1 regulatory mechanisms in plant immune responses. For example, since NPR1 transcription is not dramatically up-regulated in response to plant pathogen infections (Wang *et al.*, 2006), post-translational modifications of NPR1 regulate its localization, turnover, and functions in plant immunity (reviewed in Withers and Dong, 2016). in comparison, *CCP* transcription is dramatically induced upon pathogen challenge. Future studies on the CCP-binding transcription factors as well as the mechanism of their genetic metabolic regulation in various plants will provide evidence for copper-induced defense in plants.

## Supplementary data

The following supplementary data are available at *JXB* online.

Fig. S1. Identification of a CMV-induced copper chaperone in Arabidopsis.

Fig. S2. RT–PCR confirming the deletion of *Lys7* in the *lys7* mutant.

Fig. S3. Western blotting showing accumulation of GFP, GFP–CCP, GFP–CCP^mCBS^, and GFP–CCP^mNLS^.

Fig. S4. Confocal micrographs showing the subcellular localization of GUS–GFP–CCP, GUS–GFP–CCP^mCBS^, and GUS–GFP–CCP^mNLS^.

Fig. S5. Self-interactions of CCP derivatives and the negative controls of BiFC in *N. benthamiana* leaves.

Fig. S6. Subcellular localization of CCP in H2B–RFP transgenic *N. benthamiana* leaves treated with buffer or BCS.

Fig. S7. Subcellular localization of NbCCP, AtATX1, and AtCCH in H2B–RFP transgenic *N. benthamiana* leaves.

Fig. S8. Accumulation of *CCP* under excess copper.

Fig. S9. Accumulation of the CCP mRNA and protein in CCP overexpression lines.

Fig. S10. Identification of *ccp-1* and *ccp-2* mutant lines.

Fig. S11. BiFC analyzing the interactions of CCP and TGA2 treated with buffer or SA.

Fig. S12. Subcellular localization of TGA2 and mutants GFP, GFP–TGA2, GFP–TGA2–NT, GFP–TGA2–bZIP, and GFP–CT in H2B–RFP transgenic *N. benthamiana* leaves.

Fig. S13. GST pull-down analysis of the complex NPR1, TGA2, and CCP *in vitro*.

Table S1. Primers used in this study

eraa401_suppl_Supplementary_File001

## Data Availability

Accession numbers of genes used in this study are: Arabidopsis CCP (MN180192), ATX1 (NM_105295), CCH (NM_001339780), CCS (NM_101123); *N. benthamiana* CCP (NbCCP, XM_00959332); *S. cerevisiae* Lys7 (CCS, CP036475); and mammalian antioxidant-1 (ATOX1) (XM_004042855). The accession number of the RNA-seq data in this paper is PRJNA593673.

## Abbreviations:

CBS: copper-binding site
CCP: copper chaperone induced by pathogens
CMV: *Cucumber mosaic virus*
NLS: nuclear localization signal
