# Supplementary material for: A putative nuclear copper chaperone promotes plant immunity in Arabidopsis
Source: J Exp Bot. 2020 Aug 31;71(20):6684–96. doi: 10.1093/jxb/eraa401 (PMC7586746; doi:10.1093/jxb/eraa401)
Supplement: eraa401_suppl_Supplementary_File001 [file eraa401_suppl_supplementary_file001.pdf]

Supplementary Figures and tables

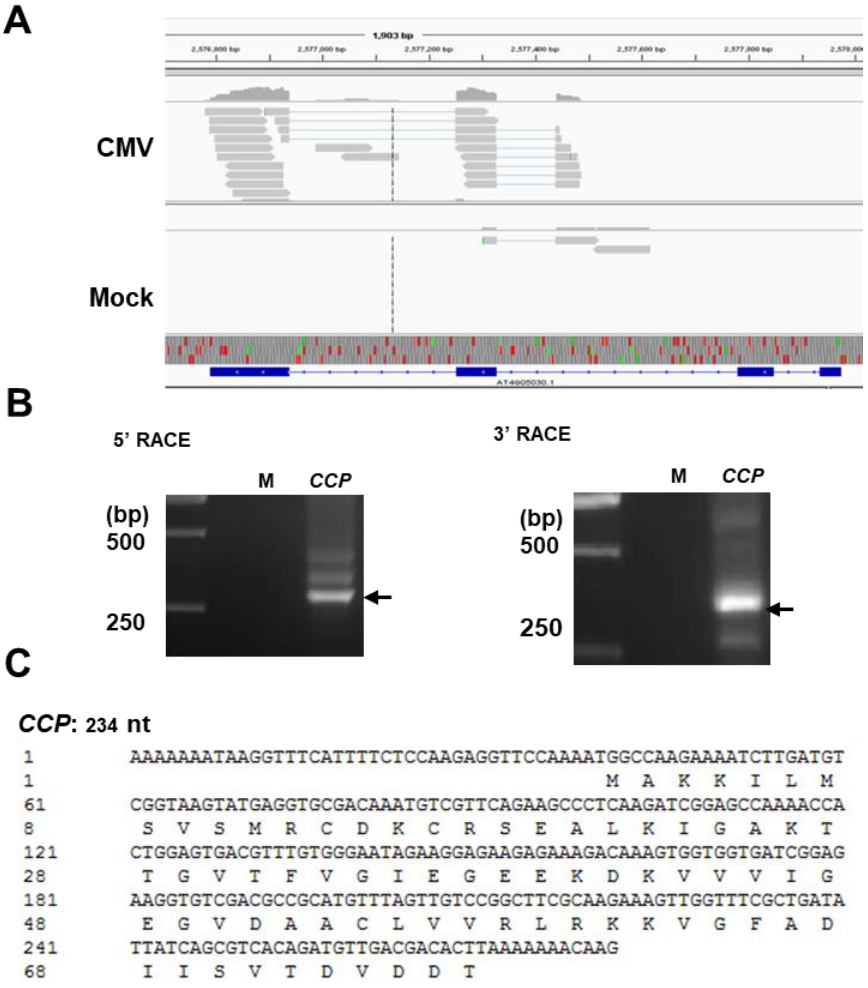

Supplementary Fig. 1. Identification of a CMV-induced copper chaperone in *Arabidopsis*. **(A)** Integrative Genomics Viewer (IGV) showing the transcriptome data analysis of the CMV-infected plants and mock plants. The bottom panel indicate the *AT4G05030* genome in the TAIR10 gene annotation, in which blue boxes and lines represent exons and introns, respectively. **(B)** Amplified PCR products of the 3' RACE and 5' RACE for sequencing. **(C)** Diagram of the nucleotide sequence and amino acid sequence of the identified CCP cDNA.

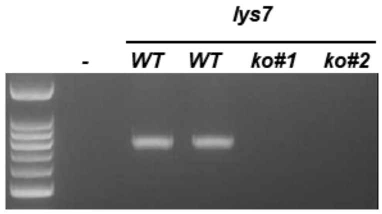

Supplementary Fig. 2. RT-PCR confirming the deletion of *Lys7* in the *lys7* mutant.

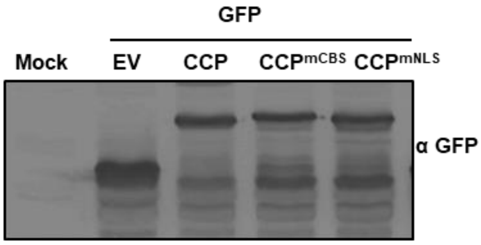

Supplementary Fig. 3. Western blotting showing accumulation of GFP, GFP-CCP, CCP^{mCBS}, and CCP^{mNLS} in infiltrated leaves of H2B-RFP-trangenic *N. benthamiana* plants at 3 dpi.

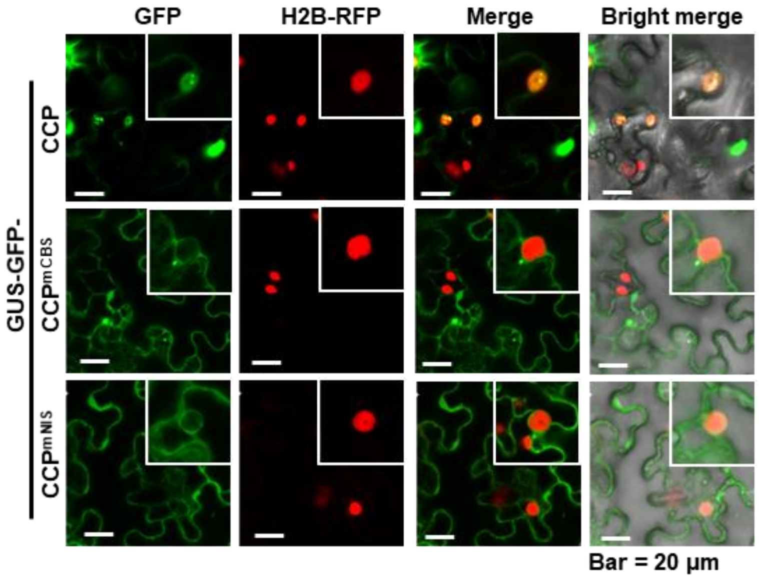

Supplementary Fig. 4. Confocal micrographs showing the subcellular localization of GUS-GFP-CCP, GUS-GFP-CCP^{mCBS}, and GUS-GFP-CCP^{mNLS} infiltrated leaves of H2B-RFP-trangenic *N. benthamiana* plants at 3 dpi.

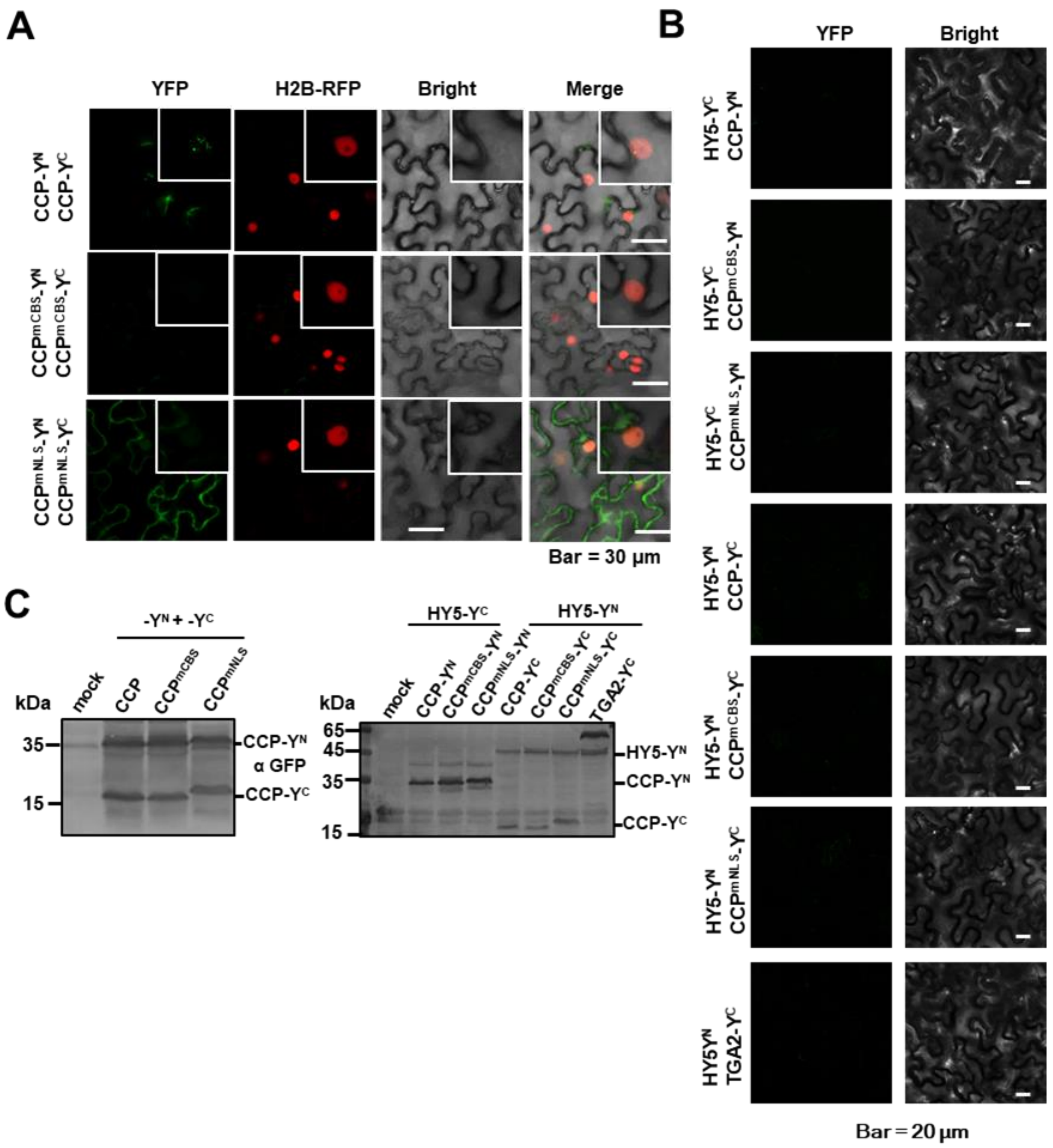

Supplementary Fig. 5. Examine the self-interactions of CCP and derivatives in *N. benthamiana* leaves. **(A)** BiFC analysis of the self-interactions of CCP, CCP^{mCBS} and CCP^{mNLS} in H2B-RFP transgenic *N. benthamiana* leaves. Bar = 30 μm. **(B)** Negative controls of BiFC assays using HY5, CCP, CCP^{mCBS}, CCP^{mNLS}, and TGA2. **(C)** Western blotting showing protein accumulation in BiFC analysis with specific GFP antibody.

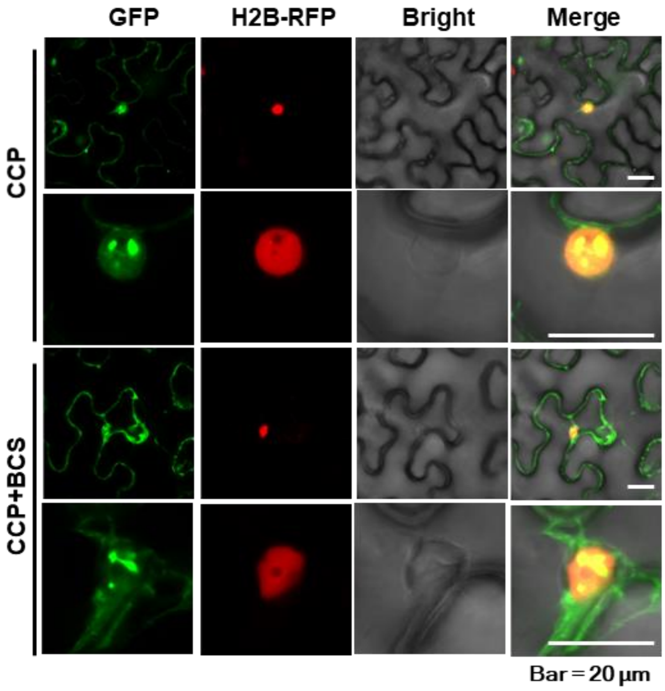

Supplementary Fig. 6. Subcellular localization of CCP in H2B-RFP transgenic *N. benthamiana* leaves treated with buffer or BCS (200 μM). Photographs were taken at 3 dpi. Bar = 20 μm

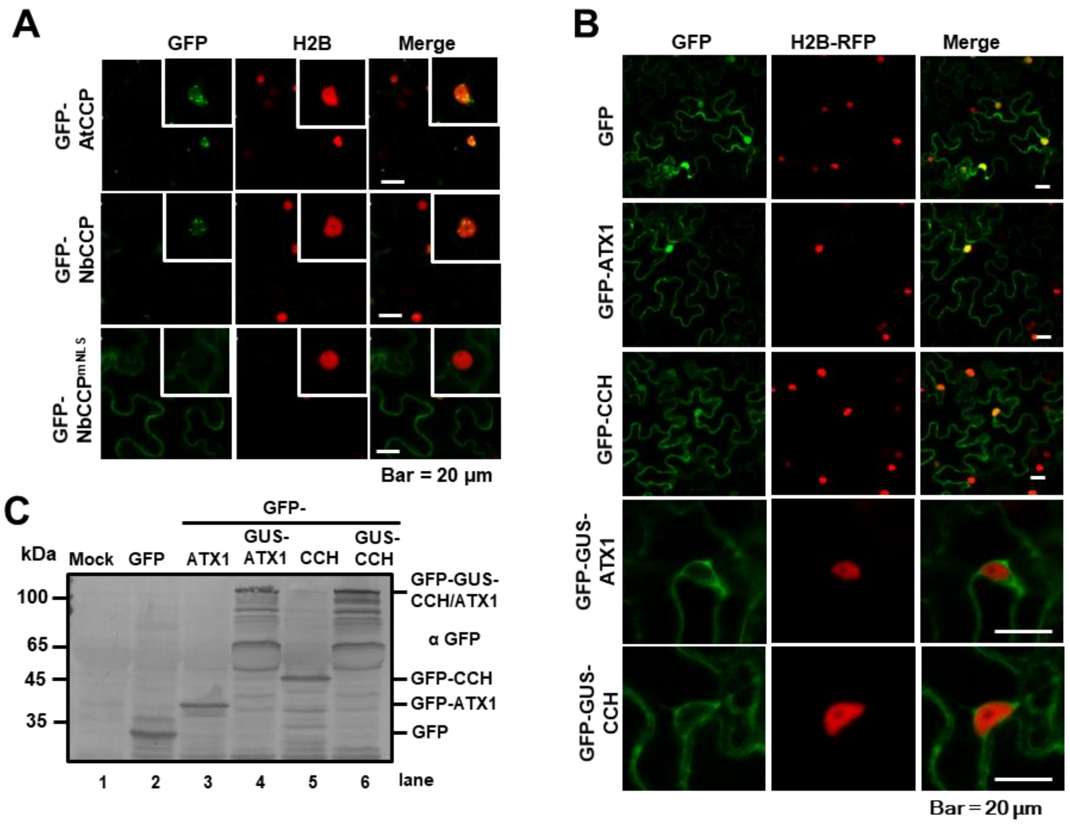

Supplementary Fig. 7. Subcellular localization of NbCCP **(A)**, AtATX1, AtCCH, GUS-ATX1 and GUS-CCH **(B)** in H2B-RFP transgenic *N. benthamiana* leaves. Photographs were taken at 3 dpi. Bar = 20 μm. **(C)** Western blotting showing accumulation of GFP, GFP-ATX1, CCH, GUS-ATX1 and GUS-CCH in infiltrated leaves of panel A and B.

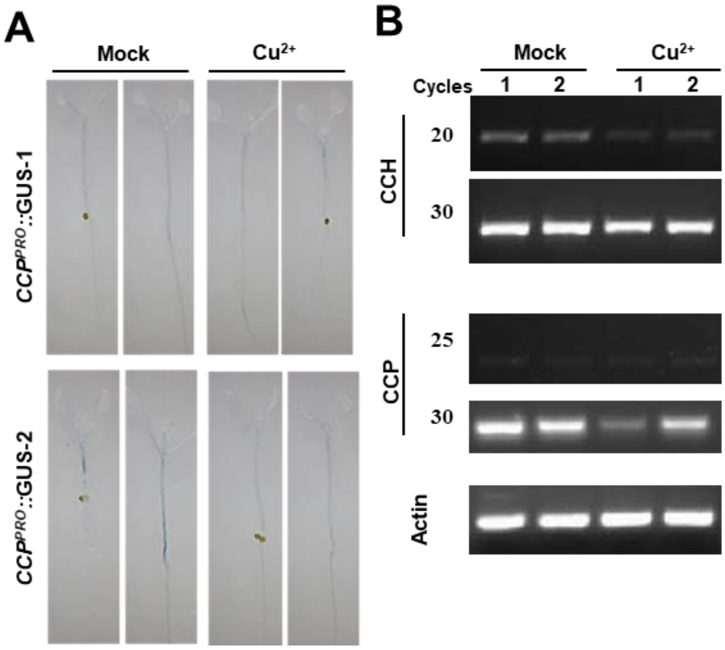

Supplementary Fig. 8. Accumulation of CCP under excess Cu. **(A)** GUS activity in two independent *CCP*^{Pro}::GUS transgenic lines treated with mock buffer or excess Cu treatment. Plants were grown on 1/2 MS for 11 d and transferred to 1/2 MS with mock buffer or CuSO4 (35 μm) for 3 d. **(B)** Semi-quantitative PCR analyzing accumulation of CCP, CCH, and actin mRNA in the plants of panel A.

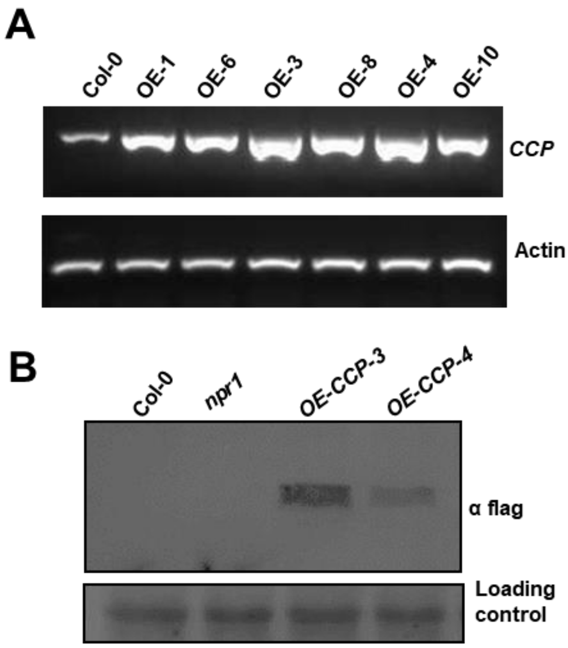

Supplementary Fig. 9. Accumulation of the CCP mRNA **(A)** and protein **(B)** in CCP overexpression lines. The Actin mRNA served as a positive control for RT-PCR. Col-0 leaves acted as a negative control. CBB staining was used as the protein loading controls.

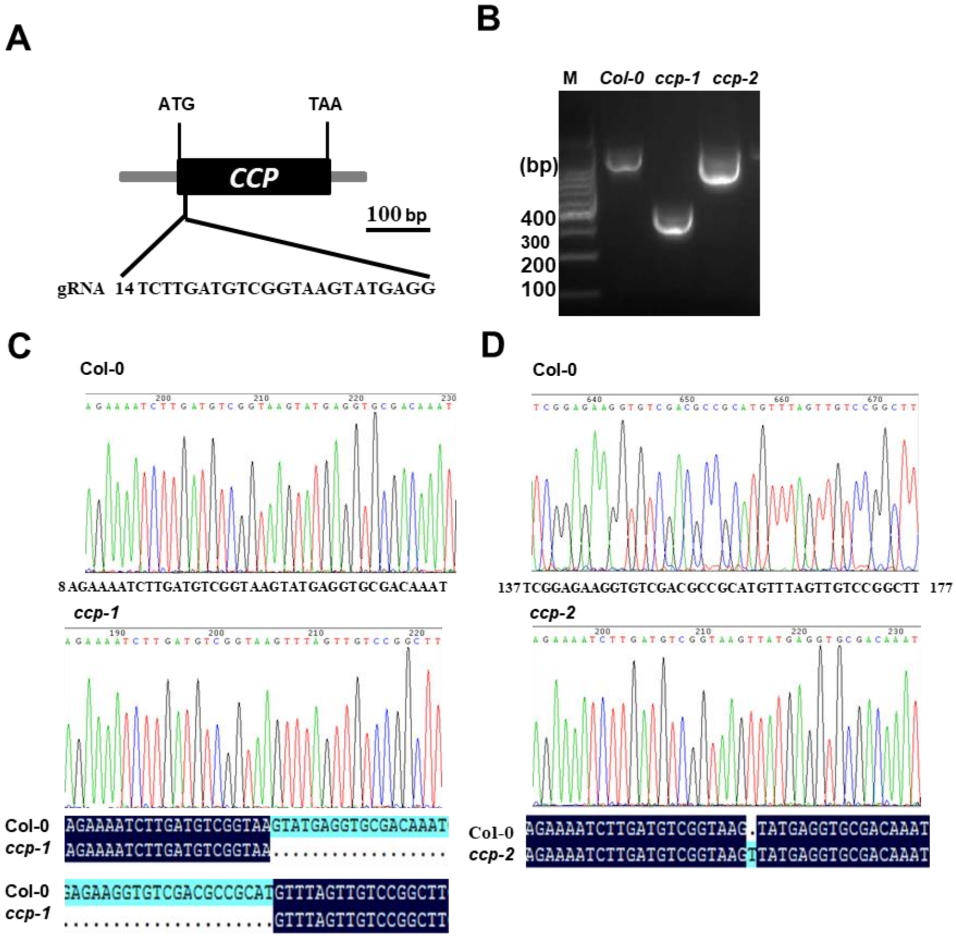

Supplementary Fig. 10. Identification of *ccp-1* and *ccp-2* mutant lines. **(A)** Diagram showing the CRISPR/Cas9-targeting site in the *CCP* cDNA. The coding sequence and untranslated regions of MDP60 are indicated by black box and gray boxes, respectively. **(B)** RT-PCR analysis of *ccp-1* and *ccp-2* mutants. Alignments between Col-0 and the *ccp-1* mutant **(C)** or **(D)** the *ccp-2* mutant, and the deletion and inserted regions are indicated by blue in the bottom panels.

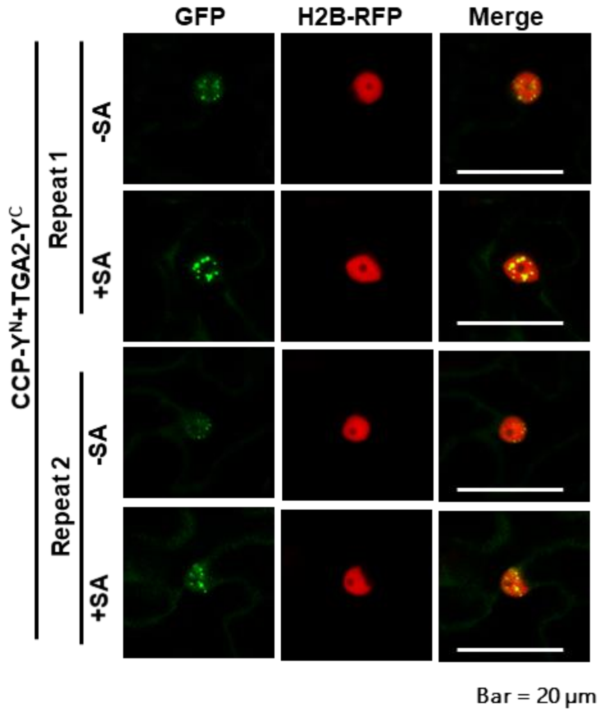

Supplementary Fig. 11. BiFC analyzing the interactions of CCP and TGA2 in epidermal cells of *N. benthamiana* leaves treated with buffer or SA (1 mM). Photographs were taken at 3 dpi. RFP, red fluorescent protein. Bar = 20 μm.

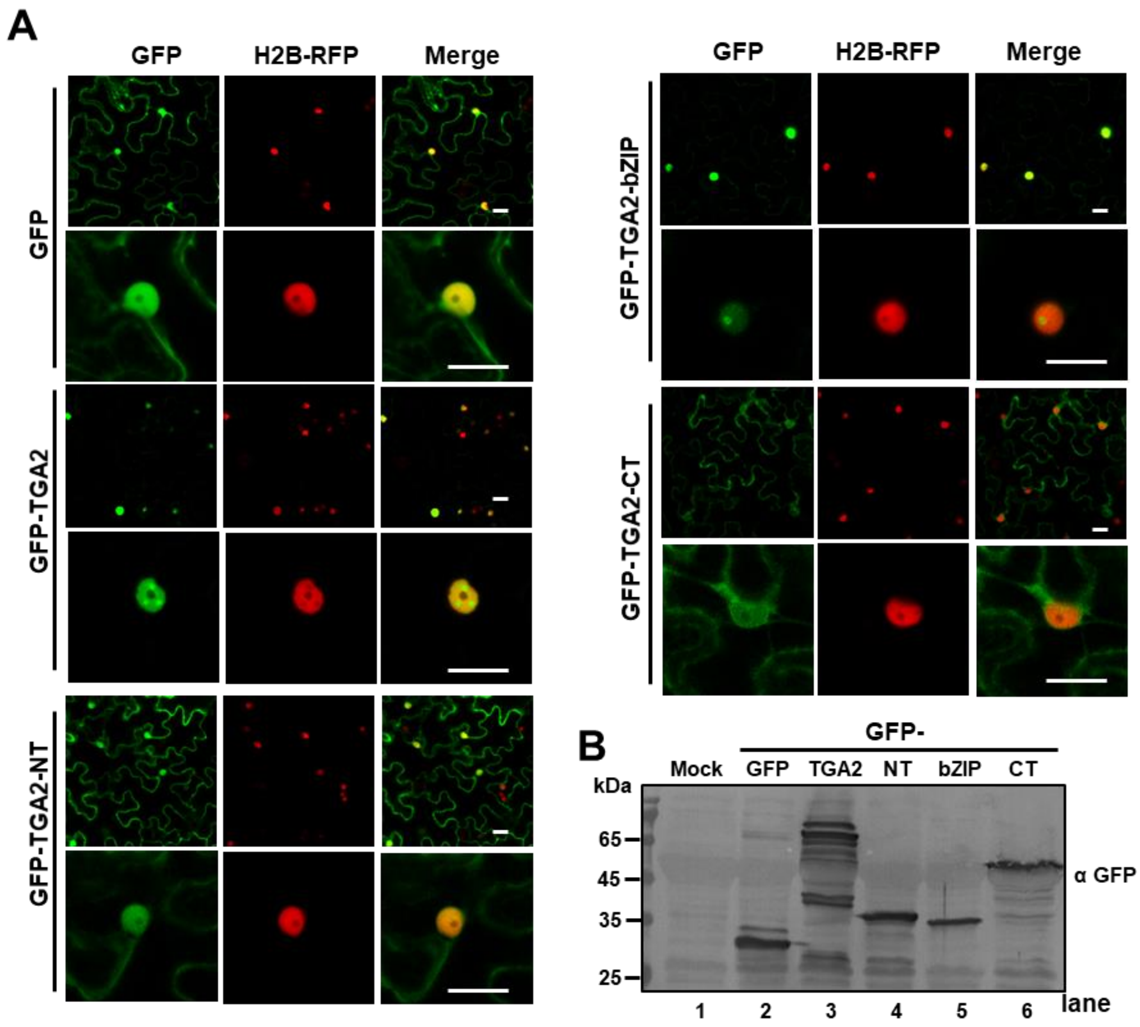

Supplementary Fig. 12. Subcellular localization of TGA2 and mutants **(A)** GFP, GFP-TGA2, GFP-TGA2-NT, GFP-TGA2-bZIP and GFP-CT in H2B-RFP transgenic *N. benthamiana* leaves. Photographs were taken at 3 dpi. RFP, red fluorescent protein. Bar = 20 μm. **(B)** Western blotting showing accumulation of GFP, GFP-TGA2, TGA2-NT, TGA2-bZIP and TGA2-CT in infiltrated leaves of H2B-RFP-trangenic *N. benthamiana* plants at 3 dpi.

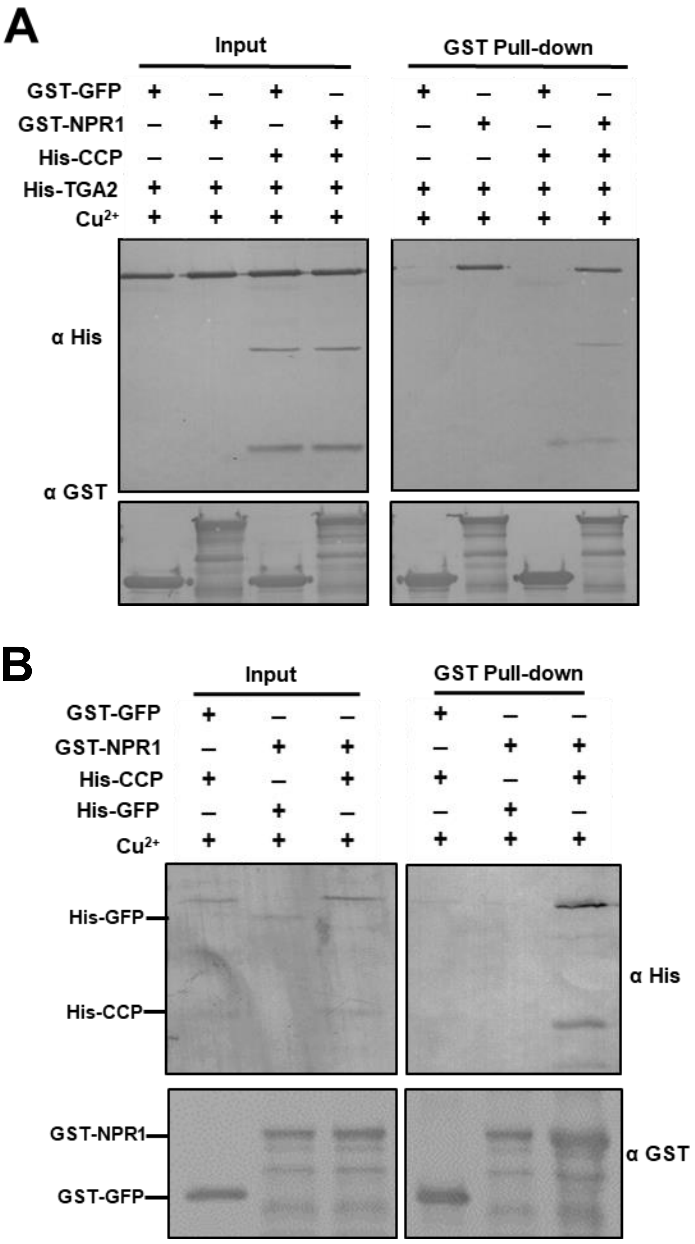

Supplementary Fig. 13. GST pull-down analysis of the complex NPR1, TGA2 and CCP *in vitro*. (A) GST-GFP or GST-NPR1 were incubated with His-TGA2 or HIS-TGA2 and HIS-CCP (with 50 μM Cu^{2+}), then immunoprecipitated with glutathione-Sepharose beads in vitro. (B) GST-GFP or GST-NPR1 were incubated with His-CCP or His-GFP for immunoprecipitation with GST beads in vitro. The Input and pull-down products were detected by western blotting analysis with anti-GST or anti-His antibodies.

Table S1 Primers used in this study

Primer[a]	Sequence (5' to 3')[b]	Notes
HC511-18TR	GGATATCTGCAGGATCCAAGCTTTTTTTTTTTTTTTTTT	For 3' race
HC511BHR	GGATATCTGCAGGATCCAAGC	For 3' race
Adaptor-F:	GTTCAGAGTTCTACAGTCCGACGATC	For 5' race
GSP-CCP-R1	AGTGTCGTCAACATCTGTGAC	For 5' race
GSP-CCP-R2	GCTGATAATATCAGCGAAAC	For 5' race
DT1-BsF	ATATATGGTCTCGATTTCTTGATGTCGGTAAGTATGGTT	Construction of *ccp* mutant by CRISPR/Cas9 system
DT1-F0	TTCTTGATGTCGGTAAGTATGGTTTTAGAGCTAGAAATAGC	
DT1-R0	AACCATGTTTAGTTGTCCGGCTTAATCTCTTAGTCGACTCTAC	
DT1-BsR	ATTATTGGTCTCGAAACCATGTTTAGTTGTCCGGCTT	
Cas9-target-F	ATACGGAAGGTTTTAGGATGAAGTAT	Detection of mutation in *ccp* mutants
Cas9-target-R	TTAAAGTGGCTAGGAAAACAGAGA	
GUS-F	CCGCTCGAGCTATGGTCCGTCCTGTAGAAACCCCAACCCG	PCR of *GUS* DNA or RT-PCR of *GUS* mRNA(*Xho1*/*Hind111*)
GUS-R	CCCAAGCTTGTTGTTTGCCTCCCTGCTGCGGTTTTTCACCG	
pGDG-CCP-F	AAAACTGCAGTCATGGCCAAGAAAATCTTGATGTCGG	RT-PCR of *CCP* mRNA (*Pst1*/*BamH1*)
pGDG-CCP-R	CGCGGATCCAGTGTCGTCAACATCTGTGACGCTG	
CCP-promoter-F1	CCCAAGCTTCACAATCCTAAAATCATCACACAAAAATCG	Construction of the promoter of the *CCP* gene(*Hind111*/*Xba1*,GUS)
CCP-promoter-R1	CTAGTCTAGAAGTGTCGTCAACATCTGTGACGCTGATAATATC	
CCP-promoter-F2	CGGAATTCCACAATCCTAAAATCATCACACAAAAATCG	Construction of the promoter of the *CCP* gene(*EcoR1*/*Xho1*,GFP)
CCP-promoter-R2	CCGCTCGAGAGTGTCGTCAACATCTGTGACGCTGATAATATC	
pMD19T-CCP-F1	CCAAGAGGTTCCAAAACGGCCAAGAAAATCTTG	Construction of the mutant of the *CCP* gene (ATG-ACG)
pMD19T-CCP-R1	CAAGATTTTCTTGGCCGTTTTGGAACCTCTTGG	
pMD19T-CCP-F2	TCGGTAAGTATGAGGGGTGACAAAGGTCGTTCAGAAGCCCTC	

pMD19T-CCP-R2	GAGGGCTTCTGAACGACCTTTGTCACCCCTCATACTTACCGA	Construction of the mutant of the *CCP* gene (CBS C13, 16G)
pMD19T-CCP-F3	CCGGCTTCGCGAAGAAGTTGGTGAAGCTGATATTATCAGCGTCACAGATGTTG	Construction of the mutant of the *CCP* gene (NLS E61R, E62R, F65R)
pMD19T-CCP-R3	CAACATCTGTGACGCTGATAATATCAGCTTCACCAACTTCTTCGCGAAGCCGG	
pMD19T-CCP-F4	CTAGCTTGTTGAGGGAAGAAGTCGGCGAAGCCAGCTTGGTGCTTGTTGATG	Construction of the mutant of the *Nb-CCP* gene (NLS)
pMD19T-CCP-R4	CATCAACAAGCACCAAGCTGGCTTCGCCGACTTCTTCCCTCAACAAGCTAG	
ccs(lys7)-F	GAATAAAGATATCTTTGCAAGGCAGAAACCCATTCGATCAGCACCGGATCCCCGGGTTAATTAA	Construction of the mutant of the *yeast ccs* strain
ccs(lys7)-R	GTTATATCTGTATTACGCTACGTTGTGCTATCTTGGATGTTGAATTCGAGCTCGTTTAAAC	
CCS(LYS7)-F	ATGACCACGAACGATACATA	Detection of the mutant of the *yeast ccs* strain
CCS(LYS7)-R	CTATTTGATGTTGTTGGCCA	
pGPD-CCP-F	CTAGTCTAGAATGGCCAAGAAAATCTTGATGTCGG	RT-PCR of *CCP* mRNA, yeast complementation(*Xba1/BamH1*)
pGPD-CCP-R	CGCGGATCCAGTGTCGTCAACATCTGTGACGC	
GST-CCP-F	TCGGATCTGGTTCCGCGTGGAATGGCCAAGAAAATCTTGAT	Construction of the N-terminal GST-fused NPR1 (*BamH1*)
GST-CCP-R	ACCACCGGAAATTCCCGGGGAAGTGTCGTCAACATCTGTGA	
GST-TGA2-F	TCGGATCTGGTTCCGCGTGGATCCATGGCTGATACCAGTCCGAG	Construction of the N-terminal GST-fused TGA2(*BamH1*)
GST-TGA2-R	ACCACCGGAAATTCCCGGGGATCCCTCTCTGGGTCGAGCAAGCC	
6×His-CCP-F	CCGGAATTCATGGCCAAGAAAATCTTGATGTCGG	Construction of the N-terminal His-fused CCP or CCP mutants (*EcoR1*/*Xho1*)
6×His-CCP-R	CCGCTCGAGTAGTGTCGTCAACATCTGTACGC	
pSPYNE-CCP-F	AGGCCTGGCGCGCCACTAGTGGATCCATGGCCAAGAAAATCTTGAT	RT-PCR of *CCP* mRNA, BiFC (*BamH1*)
pSPYNE-CCP-R	AGGTCGACAGTACTATCGATGGATCCAGTGTCGTCAACATCTGTGA	
pSPYCE-TGA2-F1	AGGCCTGGCGCGCCACTAGTGGATCCATGGCTGATACCAGTCCGAG	RT-PCR of *TGA2* mRNA, BiFC (*BamH1*)
pSPYCE-TGA2-R1	AGGTCGACAGTACTATCGATGGATCCCTCTCTGGGTCGAGCAAGCC	

pSPYCE-TGA2-R2	AGGTCGACAGTACTATCGATGGATCCCTTTTGATCCATCTTTCCCT	RT-PCR of *TGA2* (0-46 aa) mRNA, BiFC (*BamH1*)
pSPYCE-TGA2-F3	AGGCCTGGCGCGCCACTAGTGGATCCATGACTCTTCGTAGGCTTGC	RT-PCR of *TGA2* (47-95 aa) mRNA, BiFC (*BamH1*)
pSPYCE-TGA2-R3	AGGTCGACAGTACTATCGATGGATCCGACGCCCTGCTGTCTTGCTC	
pSPYCE-TGA2-F4	AGGCCTGGCGCGCCACTAGTGGATCCATGTTCATTTCAGGCACAGG	RT-PCR of *TGA2* (96-330 aa) mRNA, BiFC (*BamH1*)
pGDG-CCP-F	CCGCTCGAGCTATGGCCAAGAAAATCTTGAT	Construction of the N-terminal GFP-fused CCP or CCP mutants (*Xho1/BanH1*)
pGDG-CCP-R	CGCGGATCCTTAAGTGTCGTCAACATCTG	
pGDG-Nb-CCP-F	CCGCTCGAGCTATGCTGCAAAAGATTGTCAT	Construction of the N-terminal GFP-fused Nb-CCP or CCP mutants (*Xho1/BanH1*)
pGDG-Nb-CCP-R	CGCGGATCCCTTGATTTCATCAACAAGCACC	
pGDG-GUS-ATX1-F	CTATACAAGTCCGGACTCAGATCTATGGTCCGTCCTGTAGAAACC	Construction of the GFP-GUS-ATX1 (*BglII*)
pGDG-GUS-ATX1-R	GTCTTTAAGCATAGCTCGAGATCTTTGTTTGCCTCCCTGCTGCGG	
pGDG-GUS-CCH-F	CTATACAAGTCCGGACTCAGATCTATGGTCCGTCCTGTAGAAACC	Construction of the GFP-GUS-CCH (*BglII*)
pGDG-GUS-CCH-R	GGTCTGAGCCATAGCTCGAGATCTTTGTTTGCCTCCCTGCTGCGG	
pGDG-TGA2-F	CTATACAAGTCCGGACTCAGATCTATGGCTGATACCAGTCCGAG	Construction of the GFP-TGA2(*BglII*)
pGDG-TGA2-R	GAATTCGAAGCTTGAGCTCGAGATCTCTCTCTGGGT CGAGCAAGCC	
pGDG-NT-R	GAATTCGAAGCTTGAGCTCGAGATCTCTTTTGATCC ATCTTTCCCTTC	Construction of the GFP-TGA2-NT (*BglII*)
pGDG-bZIP-F	CTATACAAGTCCGGACTCAGATCTATGACTCTTCGTAGGCTTGC	Construction of the GFP-TGA2-bZIP (*BglII*)
pGDG-bZIP-R	GAATTCGAAGCTTGAGCTCGAGATCTGACGCCCTGC TGTCTTGCTC	
pGDG-CT-R	CTATACAAGTCCGGACTCAGATCTATGTTCATTTCAGGCACAGGAGAC	Construction of the GFP-TGA2-C T (*BglII*)
pMDC32-CCP-F	AGGCGCGCCATGGCCAAGAAAATCTTGAT	

pMDC32-CCP-R	GACTAGTAGTGTCGTCAACATCTGTGAC	Construction of the C-terminal Flag-fused CCP or CCP mutants (*Asc1/Spe1*)
pMDC32-TGA2-F	CTCTAGAGGATCCCCGGGTACCATGGCTGATACCAGTCCGAG	Construction of the C-terminal Flag-fused TGA2 (*Kpn1/Spe1*)
pMDC32-TGA2-R	GTCCATGCCACCTCCACTAGTCTCTCTGGGTCGAGCAAGCC	
LS7-F	Biotin-CCTCTTGAGAACGTCATAGAAATCTAGATT	EMSA probe
LS7-R	Biotin-AATCTAGATTTCTATGACGTTCTCAAGAGG	
npr1-F	ATGGACACCACCATTGATGGATTCGC	Detection of the mutant of *npr1*
npr1-R	CTATCCAATAGCTTCATACAAGCTTTACCAC	
AtActin2-F	GCACCCTGTTCTTCTTACCG	qRT-PCR of *AtActin2* mRNA
AtActin2-R	AACCCTCGTAGATTGGCACA	
Chip-F(a)	CCTAATGTAACATGTTATTCTAC	qRT-PCR for ChIP
Chip-R(a)	CTTTTTTATATGGAGGGAGAATCA	
Chip-F(b)	GGATAAATCTCAATGGGTGATC	
Chip-R(b)	ATGAGTATCTCTATCACTCT TG	
Chip-F(c)	TATTGACTGTTTCTCTACGTC	
Chip-R(c)	TTCTTAGTGTTTCATGCATAT	

[a] /F: forward primer; /R: reverse primer. [b] The sequence shown in lower letters is homologous to other primer as indicated in Notes column, to facilitate In-Fusion cloning;
